# Microbiome Sample Comparison and Search: From Pair-Wise Calculations to Model-Based Matching

**DOI:** 10.3389/fmicb.2021.642439

**Published:** 2021-04-07

**Authors:** Yuguo Zha, Hui Chong, Kang Ning

**Affiliations:** Key Laboratory of Molecular Biophysics of the Ministry of Education, Hubei Key Laboratory of Bioinformatics and Molecular-Imaging, Department of Bioinformatics and Systems Biology, Center of AI Biology, College of Life Science and Technology, Huazhong University of Science and Technology, Wuhan, China

**Keywords:** microbiome, search, comparison, distance-based, unsupervised, supervised

## Abstract

A huge quantity of microbiome samples have been accumulated, and more are yet to come from all niches around the globe. With the accumulation of data, there is an urgent need for comparisons and searches of microbiome samples among thousands of millions of samples in a fast and accurate manner. However, it is a very difficult computational challenge to identify similar samples, as well as identify their likely origins, among such a grand pool of samples from all around the world. Currently, several approaches have already been proposed for such a challenge, based on either distance calculation, unsupervised algorithms, or supervised algorithms. These methods have advantages and disadvantages for the different settings of comparisons and searches, and their results are also drastically different. In this review, we systematically compared distance-based, unsupervised, and supervised methods for microbiome sample comparison and search. Firstly, we assessed their accuracy and efficiency, both in theory and in practice. Then we described the scenarios in which one or multiple methods were applicable for sample searches. Thirdly, we provided several applications for microbiome sample comparisons and searches, and provided suggestions on the choice of methods. Finally, we provided several perspectives for the future development of microbiome sample comparison and search, including deep learning technologies for tracking the sources of microbiome samples.

## Introduction

**FIGURE 1:**
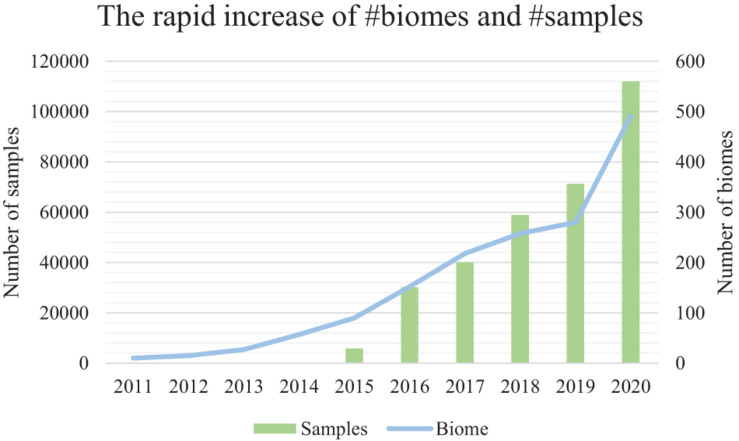
The accelerating number of microbiome samples, and the rapidly diversified biomes from where they are collected. Results are based on the assessment of the EBI MGnify database (publically available samples) from the year 2011 to the year 2020. The primary (left) vertical axis is the number of samples. The secondary (right) vertical axis is the number of biomes. EBI MGnify database is pubilc and available.

Microbiome samples are accumulating at an accelerating rate, representing microbial communities from every niche (biome) of the human body as well as other host organisms, environments, and ecological biomes (Mitchell et al., 2020; Figure 1). Comparison of microbiome samples, as well as searching for microbial community samples among a pool of millions of samples, thus becomes increasingly important for the discovery of similar samples and their intricate relationships (Knights et al., 2011; Shenhav et al., 2019), the tracking of possible sample origins (Lax et al., 2014), and the mining of key species and functional genes (Che et al., 2019).

Microbial source tracking (MST) has a very broad application area, including environment science, public health, food science, and forensics, etc. (Fu and Li, 2014; Lax et al., 2014; Henry et al., 2016; Metcalf et al., 2016; Gu et al., 2020; McHugh et al., 2020). For example, many MST studies have focused on determining sources of fecal contamination in waterways, and they are also providing the scientific community with tools for tracking both fecal bacteria and food-borne pathogens contamination in the food chain (Li et al., 2018; Han et al., 2020). Approaches for MST are commonly classified as library-dependent methods (LDMs) or library-independent methods (LIMs). Unsupervised tools and distance-based tools are usually LDMs, such as FEAST (Shenhav et al., 2019) and UniFrac (Lozupone et al., 2011). On the other hand, supervised tools are mainly LIMs, such as Random Forest and the recently developed ONN4MST^1^. In general, LDMs and LIMs can both achieve good performance for MST with a small number of microbial community samples (usually from a handful to dozens of samples) and a few biomes (usually no more than 10 biomes). However, LIMs can also perform well when source tracking with thousands of samples and hundreds of biomes, but it is difficult for LDMs to deal with such situations due to limitations of accuracy and efficiency.

However, as the number of microbiome samples easily exceeds tens of thousands in a medium-sized data collection, the efficiency and accuracy of sample comparison and search becomes a critical bottleneck (Figure 1). Besides, there are millions of samples from the rapidly diversified biomes (biome size is about 100–300 samples) in public databases (i.e., MGnify) (Figure 1). The rapidly increasing number of samples from various niches on the planet has thus created a difficult hurdle for knowledge discovery from these samples (Mitchell et al., 2020).

## Current Methods for Microbiome Sample Comparison and Search

**FIGURE 2:**
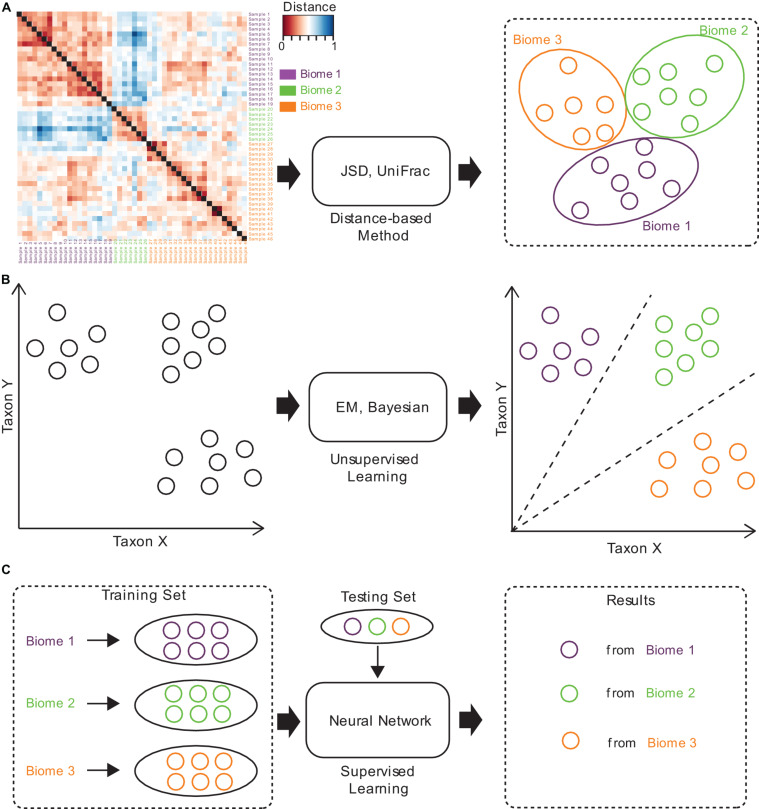
The brief computational workflows for microbiome sample comparison and search based on **(A)** distance-based methods, **(B)** unsupervised methods, and **(C)** supervised methods.

Several approaches have been established for microbiome sample comparison and search. These approaches include the methods based on pair-wise calculations of sample distances (distance-based methods), unsupervised methods, and supervised methods (Figure 2). These methods are different in the utilization of the microbiome data (species abundance information, species phylogenetic information, or both), the preprocessing of the microbiome data (feature selection), the underlining computational models (distance calculation, model-free unsupervised calculation, or model-based supervised learning), and the utilization of the computational resources (multi-thread, memory-efficient optimization, etc.) (Table 1).

**TABLE 1:** Computational resources and data utilization differences among current methods for microbiome sample comparison and search.

Method	Methods in use	Representative tools	Pre-process of the data	Computational model	Utilization of computational resources
Distance-based	Pair-wise sample distances	JSD (Lin, 1991)	Could apply feature selection before source tracking	Pair-wise distance calculation	No optimization
		UniFrac (Lozupone et al., 2011)			
		Meta-Storms (Su et al., 2012)			
		Meta-Prism (Zhu et al., 2020)			
Unsupervised	Bayesian	SourceTracker (Knights et al., 2011)	No feature selection	Model-free unsupervised calculation	No optimization
	EM	FEAST (Shenhav et al., 2019^)^			
Supervised	Ensemble learning	Random Forest (Roguet et al., 2018)	Could apply feature selection before source tracking	Model-based supervised learning	Multi-thread, memory-efficient optimization
	Neural Network	ONN4MST (https://github.com/HUST-NingKang-Lab/ONN4MST)			

Traditional methods for microbiome sample comparison are based on the pair-wise calculation of sample distances (Figure 2A), and such methods depend heavily on the presence of species and their relative abundances for individual samples, no matter whether weighted or unweighted scoring functions are used (Lin, 1991; Lozupone et al., 2011). For example, Jensen-Shannon divergence (JSD) is a distance-based method, which estimates the JSD distance between samples. Specifically, for a pair of samples, it calculates the distance by computing the distribution similarity of two abundance tables. The procedure of JSD can be represented by the following formula:

$$D_{X\,Y}=\frac{\sum_{1}^{N}{\left(X_{i}-Y_{i}\right)}^{2}}{N}$$

where *X* and *Y* each represent a microbiome sample, *D*_*XY*_ is the JSD distance between the two samples, *i* is a specific species, *X*_*i*_ is the relative abundance of species *i* in sample *X*, *Y*_*i*_ is the relative abundance of species *i* in sample *Y*, and *N* is the number of species detected in the two samples. However, such methods have not considered the specific features of a set of samples from similar niches, and the distance-based methods have a binomial increase of time cost with the increase of the number of samples.

Unsupervised methods for microbiome sample comparison are based on profile-based statistical models, either the Bayesian model (Knights et al., 2011) or the Expected-Maximization (EM) model (Shenhav et al., 2019), for more accurate comparison and search (Figure 2B). Unsupervised methods usually need samples to be converted into a feature table, which is then used for statistical inference of similarities. Unsupervised methods are usually more accurate than distance-based methods, while the speeds are similar. For example, SourceTracker (Knights et al., 2011) is an unsupervised method, which requires consideration of all possible assignments of the test sample sequences to the different source environments (such as gut, oral, skin, soil, and water). SourceTracker considers each microbiome sample as a set of sequences mapped to taxa, where each sequence can be assigned to any one of the source environments. Then, it conducts Gibbs sampling to estimate the proportion of bacteria from source environments. However, since unsupervised methods still do not consider the specific features of a set of samples from similar niches, their tolerance to noisy signals in samples remains poor, and would lead to biased mismatches.

Supervised methods are also referred to as model-based methods (Figure 2C). The models used in the supervised methods include the Random Forest (RF) model (Roguet et al., 2018), as well as the neural network (NN) model (see text footnote 1). Since supervised methods take into consideration the specific features of a set of samples from similar niches, they are tolerant to noises in samples, but can still discover similar samples even from distant niches. Moreover, model-based methods are magnitudes faster than distance-based and unsupervised methods, which is mainly because all sample searches are based on the pre-built model. Therefore, supervised methods are suitable for large-scale comparisons and searches, and are superior in both accuracy and speed.

Nevertheless, all three approaches, namely distance-based calculation and unsupervised matching, and supervised matching, have their specific application scenarios: for a few samples, ranging from a few tens to several hundreds, both distance-based calculation and unsupervised matching approaches could provide quick and accurate sample comparisons, provided that these samples were from a handful of niches (biomes). Thus, these two approaches are suitable for a quick comparison of samples in hand. However, if the number of samples exceeds a thousand, or samples have a high level of heterogeneity (from many biomes), or some samples are very similar, then the supervised matching approach would be more accurate.

In terms of efficiency, both time and memory costs are important. Provided that all pre-processes are already done, the supervised matching method is superior to other methods. However, when considering memory costs, then distance-based methods are very effective since they do not need to store any models for comparison. The supervised matching method is also good since all comparisons are based on pre-built models, and it is better than distance-based methods when the number of samples is larger than a thousand. Thus, for large-scale sample comparisons, the supervised matching method is better than other methods in efficiency as well. Here we should note that the supervised matching approach could achieve superior accuracy and speed for large-scale comparisons and searches, but at the cost of model building: when the number of samples considered in the model exceeds one million, the model building process would take more than 1 day.

Microbiome sample comparison is not only about the comparison and searching of sample purpose, but also for knowledge discovery from microbiome big-data. One kind of such knowledge is about remote similarities between samples, especially similar samples from different niches. Such similarity patterns would always lead to the discovery of patterns that are common for certain environmental conditions. Another set of information that could be discovered is the microbiome samples of special functions, such as adaptation to specific conditions. The third set of knowledge is about the differences among samples from similar niches, which could always reflect the evolutionary patterns of communities.

## Complexity of the Source Tracking Task

**FIGURE 3:**
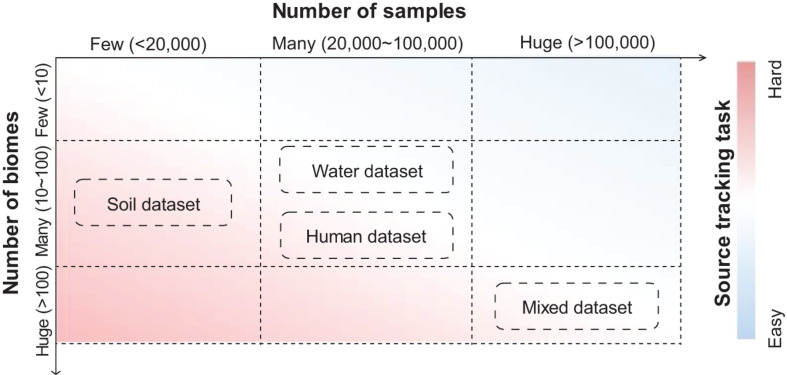
Datasets with varied complexities have provided source tracking tasks with different difficulties. Soil datasets contain samples mainly from soil biomes. Water datasets contain samples mainly from water biomes. Human datasets contain samples mainly from human biomes. Mixed datasets contain samples from all biomes in MGnify. Estimations of the number of biomes and samples are based on the MGnify database in the year 2020.

The complexities of source tracking tasks are heavily dependent on the number of samples and the number of biomes in a dataset. Source tracking tasks are usually easy when there are many samples from a few biomes, while they are usually hard when there are samples from many biomes (Figure 3). A typical example of a dataset with low complexity is human gut samples from different continents, in which a simple Random Forest method could differentiate samples from these continents fairly accurately (Yatsunenko et al., 2012). A typical example of a dataset with high complexity is for “open search” samples from a pool of millions of samples from hundreds of biomes. Those datasets, such as those with thousands of samples from the soil environment or water environment, as well as those with tens of thousands of samples from the human body, are of medium complexities.

These datasets are introduced by a relevant study (Zha et al., 2020). In their study, Zha et al., 2020 collected 125,823 samples from the MGnify database (mixed dataset), including 53,553 samples from human environments (human dataset), 27,667 samples from water environments (water dataset), and 11,528 from soil environments (soil dataset), and details (also include download links) about these mentioned datasets can be learned through the Supplementary Material of their study. Indeed, these datasets comprise samples from different niches, which are representative of high-quality samples in public resources.

The fundamental reason for such a complex source tracking task is that when there are many samples but few biomes, differences between biomes can be extracted with high fidelity, making it relatively easy to distinguish samples from different biomes. On the other hand, when the number of biomes is large but the number of samples is small, it is difficult to clearly distinguish the biomes due to the lack of sample taxonomic characteristics.

Datasets with different complexities could serve well for bench-marking and comparison of various source tracking methods. A complex dataset (dataset containing samples mostly from “soil” biomes; Mitchell et al., 2020; see text footnote 1), which covers less than 20,000 samples from more than 10 biomes, could provide the opportunity to assess the potential extremes of accuracy for these methods. Meanwhile, a huge dataset (dataset containing samples from “human,” “soil,” and “water” biomes; Mitchell et al., 2020; see text footnote 1) which covers more than 100,000 samples, could provide the opportunity to assess the efficiency of both time and memory for these methods.

## Distance-Based Methods

Distance-based methods, also known as similarity score-based methods or difference score-based methods, represent the first generation of attempts for microbiome sample comparison and search. These methods include JSD, Bray-Curtis distance, the UniFrac series, and the Meta-Prism series. These methods differ in the utilization of microbiome data (species abundance information, species phylogenetic information, or both), and the optimization of computational resources (multithreading, memory-efficient optimization, etc.).

Among distance-based methods, some consider phylogenetic relationships of species in samples and some do not. JSD (Lin, 1991) is a typical method that does not consider the phylogenetic relationships of species in samples. Bray-Curtis (Beals, 1984) distance is another typical distance-based method but still does not consider the phylogenetic relationships of species in samples. The UniFrac, Fast UniFrac, and Striped UniFrac methods are commonly used methods that do consider the phylogenetic relationships of species in samples (Lozupone et al., 2011). Meta-Storms and Meta-Prism methods are also widely used methods that do consider the phylogenetic relationships of species in samples. Meta-Storms and Meta-Prism methods are different from the UniFrac series mainly due to the different scoring functions used (Su et al., 2012; Zhu et al., 2020): while UniFrac calculates the similarities between samples, the Meta-Storms and Meta-Prism methods calculate the distances between samples, and the detailed scores are also different. Meta-Prism has also been optimized in a sample data storage format and in multithreading computation for better processing speed (Zhu et al., 2020).

Distance-based methods are most suitable in situations under which we need to quickly identify the similarities among samples on a global scale, while they are not very suitable for scrutinizing the tiny differences among samples, largely due to the imprecise nature of the distance-based methods.

However, distance-based methods are usually slow. For example, searching for a sample against a database of more than one million samples takes JSD a few minutes, while UniFrac takes tens of minutes. On the other hand, all distance-based methods are optimized in sample storage as well as multithreading computation for better efficiency. Meta-Prism is one of the most efficient methods for sample searches by optimizing computational thread and memory. For example, Meta-Prism could complete a search in less than 1 s by only using 1 GB of memory when searching a sample against a database of more than one million samples. Striped UniFrac could reach a similar speed but requires more than 10 GB of memory.

## Unsupervised Methods

Distance-based methods could be used for microbiome sample comparisons and searches, but are not very accurate. Thus, one might consider unsupervised methods to solve this same problem with higher accuracy. Unsupervised methods for microbiome sample comparison and searches include Bayesian and EM (expected maximization) methods, etc.

The Bayesian method and EM method are unsupervised methods that can accurately compare microbiome samples, taking into consideration the phylogenetic relationships of species in the community. SourceTracker is a typical sample search method based on the Bayesian algorithm (Knights et al., 2011). SourceTracker’s distinguishing features are its direct estimation of source proportions, and its Bayesian modeling of uncertainty about known and unknown source environments. The Bayesian approach requires consideration of all possible assignments of the test sample sequences to the different source environments, SourceTracker has explored this joint distribution using Gibbs sampling, a technique widely used in the exploration of complex posterior distributions.

The EM method is more efficient than the Bayesian method for sample source tracking. FEAST is a typical sample search method based on the EM algorithm (Shenhav et al., 2019). The statistical model used by FEAST assumes each sink is a convex combination of known and unknown sources. FEAST has two hyperparameters: the convergence threshold and the maximum number of iterations. The convergence threshold determines the minimum value difference between the sum of all sources’ probabilities and 1 in the M step (maximization). The number of iterations determines the maximum number of rounds in the E step (expectation) runs.

Unsupervised methods are most suitable when there are a few samples for quick comparison, or the dataset to be searched is very small so that model-based methods are not necessary. However, unsupervised methods do not consider the specific features of a set of samples from similar niches, its tolerance to noisy signals in samples is not high, thus would lead to biased mismatches. Again, faced with millions of samples for source tracking, the unsupervised methods fall short in speed and memory utilization (Table 2).

**TABLE 2:** The advantages and disadvantages of current methods for microbiome sample comparison and search.

Method	Methods in use	Suggested sample size	Suggested biome size	Search accuracy	Time cost (s/query)	Memory cost (GB/query)
	
	Representative tools					
Distance-based	JSD	1∼10^5^	1∼10	0.5∼0.8	10^1^∼10^2^	10^0^∼10^2^
	UniFrac	1∼10^5^	1∼10	0.6∼0.8	10^0^∼10^1^	10^0^∼10^2^
	Meta-Storms	1∼10^5^	1∼10^2^	0.5∼0.8	10^–1^∼10^0^	10^1^∼10^2^
	Meta-Prism	1∼10^5^	1∼10^2^	0.5∼0.8	10^–1^∼10^0^	**10**^–1^**∼10**^0^
Unsupervised	Source tracker	1∼10^4^	1∼10^2^	0.8∼0.9	10^2^∼10^4^	10^0^∼10^1^
	FEAST	1∼10^5^	1∼10^2^	0.8∼0.9	10^1^∼10^3^	10^0^∼10^2^
Supervised	Random forest	**1∼10**^6^	1∼10^3^	**>0.9**	**10**^–2^**∼10**^–1^	10^0^∼10^1^
	ONN4MST	**1∼10**^6^	1∼10^3^	**>0.9**	**10**^–2^**∼10**^–1^	10^0^~10^1^

**Values in bold indicate the best performance among these methods in accuracy and efficiency. Estimations of search accuracy and efficiency are based on the results of recent studies (Shenhav et al., 2019; Zha et al., 2020).**

## Supervised Methods

Though unsupervised methods are accurate for microbiome sample comparison and searches, it is easy to think of model-based methods as solving the same problem with higher accuracy and speed. However, it was not until recently that supervised methods were realistic, largely due to the number of samples accumulated.

The Random Forest method (Roguet et al., 2018) is a classification method that calculates the highest probability from which class the query sample belongs. The Random Forest algorithm creates decision trees on data samples and then obtains a prediction from each of them and finally selects the best solution through voting. It is an ensemble method that is better than a single decision tree because it reduces the over-fitting by averaging the result. With the accumulation of microbiome samples, more decision trees could be created for the Random Forest algorithm and thus be more accurate in MST.

The neural network (NN) approach is a typical supervised approach for sample comparison and searches. A neural network model for MST can generally be divided into three parts. The input layer receives microbial community sample data, the hidden layer would learn the distribution of all samples, and the output layer gives the contribution of each biome for samples. The ontology-aware neural network (ONN) approach (see text footnote 1) is an improved supervised approach that takes into consideration not only the phylogenetic relationships of species in the community, but also the hierarchical relationships of biomes (Mitchell et al., 2020)^2^. Additionally, the ONN approach employs a hierarchical architecture of the neural network that fits in with the biome ontology and thus can source track microbial community samples with high accuracy in every layer of biome ontology. Moreover, the ONN approach also shows superiority in time and memory cost. However, it should be noted that current supervised methods are heavily dependent on the completeness of the sample collections for building the model, which is an inherited limitation of supervised approaches.

In summary, all three approaches have advantages and disadvantages (Table 2). Since these three approaches have specific niches in which they are most suitable for application, there is constant improvement in all of these three approaches. For example, the recently developed Striped UniFrac (McDonald et al., 2018) has largely improved the search efficiency of the UniFrac series of tools. Despite these ongoing developments, supervised methods have shown superior performances in terms of both accuracy and speed for large-scale microbiome sample comparison and searches.

## Assessment of Different Methods on a Standard Benchmark Dataset

To compare the accuracy and efficiency of these three types of methods, we conducted an assessment on a standard benchmark dataset. We used the subset of EMP (Earth Microbiome Project) in the CAMDA challenge competition (year 2019)^3^ for the following evaluation. There were 3,043 earth microbiome samples from 21 soil associate biomes, out of which eight biomes contained more than 100 samples (Supplementary Table 1). We randomly selected 800 samples from these eight biomes (100 samples per biome). Among these 800 samples, 90% (720 samples) were used for sources (training samples) and the remaining 10% (80 samples) were used for sinks (testing samples), and this training/testing data separation procedure was repeated eight times. We applied three representative tools for comparison, including the distance-based method (i.e., Bray-Curtis), unsupervised method (i.e., FEAST) and supervised method (i.e., neural network).

Results have shown that the supervised method has superior performance compared to the other two methods in accuracy and efficiency. For instance, the simple neural network achieved an accuracy of 0.900 with a time cost of 11 s, while though the unsupervised method and distance-based method took much longer, their accuracies were much lower (Table 3).

**TABLE 3:** The comparisons of current methods for microbiome sample searches’ accuracy and efficiency.

Method	Representative tools	Accuracy (MEAN ± STD)	Time cost^*a*^	Memory cost^*b*^
Distance-based	Bray-Curtis	0.525 ± 0.323	34 s	0.21 GB
Unsupervised	FEAST^*c*^	0.775 ± 0.238	4 h	0.78 GB
Supervised	Neural Network^*d*^	0.900 ± 0.050	11^*e*^ s	0.53 GB

*^*a*^Running time on a single core.*

*^*b*^Maximum memory cost when programs running.*

*^*c*^Due to its low efficiency, FEAST is tested on 80 source samples only.*

*^*d*^A simple neural network with two fully connected layers as hidden layers and ReLU as the activation function.*

*^*e*^Query time only (excluding training time). Accuracy is measured by dividing the number of samples with correctly predicted source biomes, over the number of all samples.*

## Applications of Microbiome Sample Comparison and Search

Microbiome sample comparison and search methods have a broad application area (Table 4), especially in sample source tracking.

**TABLE 4:** Current applications of microbiome sample comparison and searches.

	Example	Description	References
Hospital sink	Microbial contamination detection in built environment	By using SourceTracker, researchers found that draft genomes of potential human pathogens observed on a kitchen counter could be matched to the hands of occupants.	Lax et al., 2014
Public health	COVID-19 source tracking	By using the Random Forest method, researchers selected five biomarkers for distinguishing COVID-19 patients from healthy controls, results showed a high accuracy with an area under the curve (AUC) up to 0.89.	Gu et al., 2020
Forensic studies	Microbial community assembly and metabolic function during mammalian corpse decomposition	Researchers sampled the skin and gravesoil associated with four decomposing human bodies, as a consequence, microbial succession during decomposition appears to be a predictable process that has implications for biogeochemical cycling and forensic science.	Metcalf et al., 2016
Food chain	MST methods in an application to understand the source and transmission of food-borne pathogens	Researchers discussed microbial source tracking methods application for identifying sources of bacterial contamination in the food chain in three main aspects: water for agriculture and aquaculture, food animals in a farming environment, and food products in post-harvest processing	Fu and Li, 2014
Agriculture research	Tracking the dairy microbiota from farm bulk tank to skimmed milk powder	This study set out to use molecular methods to provide an important description of the microbiota of a food processing pipeline by tracking the microbiota of raw milks on farms to a final skimmed milk powder.	McHugh et al., 2020
Environmental research	Evaluation of SourceTracker for the assessment of fecal contamination of coastal waters	Application of SourceTracker to recreational beach samples identified treated effluent as a major source of human-derived fecal contamination, present in 69% of samples.	Henry et al., 2016
Conservation biology	Linking watershed modeling and bacterial source tracking to better assess *E. coli* sources	This study proposed a model to identify critical source areas of *E. coli* in mixed land uses in south Texas, and results showed that wildlife contribution is the major source of *E. coli* in streamflow, and may remain to be significant after land use change with urbanization.	Jeong et al., 2019

In all health systems, tracking the source of a hospital sink sample is an important issue for the prevention of possible infectious diseases (Lax et al., 2014; Brown et al., 2019). Identification of possible contamination from hospital sinks and other niches has thus become an urgent need. SourceTracker has been applied in several hospital sink sample source tracking applications to identify human and animal contamination with high confidence (Lax et al., 2014; Brown et al., 2019). FEAST reanalyzed some of these datasets, confirming the contamination sources qualitatively, yet with different quantitative assessment results (Lax et al., 2014; Shenhav et al., 2019).

In public health research, sample source tracking is also key for fast and accurate action against infectious diseases^4^. With the ever-increasing size of cities, possible pollution from city residents to the environment, especially fecal contamination to the environment, has become a critical public health issue (Henry et al., 2016; Staley et al., 2018). SourceTracker has been applied for these datasets and identified the sources of fecal contamination with high fidelity.

During the recent outbreak of COVID-19, microbiome sample source tracking also played an important role in finding the relationships among patients. It has already been reported that COVID-19 patients have profoundly different gut microbiota compared with healthy individuals (Gu et al., 2020). By using the Random Forest method, researchers selected five biomarkers for distinguishing COVID-19 patients from healthy controls, results showed high accuracy with an area under the curve (AUC) up to 0.89 (Gu et al., 2020).

In forensic studies, corpse sample source tracking is a critical issue (Metcalf et al., 2016). Based on microbiome samples, it has already become possible for forensic scientists to identify the microbial sample coming from important suspects/sources based on microbial community dissimilarities (Carter et al., 2020). Researchers sampled the skin and gravesoil associated with four decomposing human bodies, as a consequence, microbial succession during decomposition appears to be a predictable process that has implications for biogeochemical cycling and forensic science (Metcalf et al., 2016).

In a food chain, sample source tracking could also help for the detection of possible contaminates (Fu and Li, 2014). The ability to trace fecal indicators and food-borne pathogens to the point of origin has major ramifications for food industries, food regulatory agencies, and public health. Such information would enable food producers and processors to better understand sources of contamination and thereby take corrective actions to prevent transmission. Microbial subtyping and source tracking have also been used to investigate the transmission of other major zoonotic pathogens from pre- to postharvest food animals. *Salmonella* has been associated with poultry meat and egg products, and these bacteria are capable of colonizing in live poultry in their intestinal tracts. Source tracking and horizontal transmission pathways of Salmonella serovars were delineated in a turkey production environment (Nayak and Stewart-King, 2008).

In agriculture research, sample source tracking is a powerful tool against pollutants (McHugh et al., 2020). This study set out to use molecular methods to provide an important description of the microbiota of a food processing pipeline by tracking the microbiota of raw milks on farms to the final skimmed milk powder. With the routine implementation of these source tracking methods, understanding the causes that lead to different species being dominant in the final product can be determined and lead to informed decisions regarding product fate, in turn leading to increased food safety, reduced risk, and reduced economic losses.

In environmental research, sample source tracking could help toward better environmental protection (Hagedorn et al., 2011), as well as for the protection of public health indirectly (Harwood et al., 2014). SourceTracker applied a Bayesian model to derive proportions of sources within sink samples. However, it was recognized that the model reported high variability in estimates for sources present at low concentrations. This limitation is a potential problem for the detection of human fecal contamination within coastal waters where levels are often low but still pose public health risks. FEAST applied the EM algorithm to derive proportions of sources within sink samples, it could get a high resolution when faced with problems in pollution detection, and thus protect us from potential public health risks. Besides, in conservation biology research, sample source tracking could also help researchers to better understand the contributing sources of bacterial contamination, assessing the management of critical sources (i.e., wildlife animals), and reducing concentrations of pollution indicator bacteria in wildlife environments (Byappanahalli et al., 2015; Jeong et al., 2019).

All in all, computational method microbiome sample comparison and searches could have a very broad application area, including applications related with the host-associated microbiome, as well as the environmental microbiome. This again emphasizes the importance of fast, accurate, and reliable methods for microbiome sample comparison and searches, especially those related with sample source tracking.

## Discussion and Conclusion

Pronounced applications depend on accurate and fast microbiome sample comparison and searches. The microbiome sample comparison and searches, especially faced with millions of microbiome samples, has thus become one of the most important problems in the microbiome research field.

Three main approaches, namely the distance-based method, unsupervised method, and supervised method, are presently commonly used for microbiome sample comparison and searches. They represent the paradigm shift in the area, and are in line with the microbiome big-data. Though distance-based methods, such as Striped UniFrac (McDonald et al., 2018) and Meta-Prism^5^, have been optimized to the minimum usage of memory and near-optimal speed, their accuracies are lagging behind machine learning methods, especially when the number of samples to be searched against exceeds tens of thousands up to millions. Among the machine learning methods, model-based supervised methods are superior to unsupervised methods, both in terms of accuracy and efficiency.

How far are we to the final solution to the problem of microbiome sample comparison and searches? In regard to accuracy, not far if we are using supervised methods based on pre-built models. However, this is not realistic if a sample from totally new niches is sent in for a search. Therefore, the combination of several approaches might still have its usage for a long time. In regard to efficiency in time and memory usage, we can confidently say that current methods have reached a limit, provided that current parallel computation and GPU computation have no revolutionary update. This is said based on the fact that there is very little margin between the time and memory usage for a sample search, compared with the cost of only conducting I/O of the sample, in regard to the actual or ratio of the time/memory space used (Table 2).

Currently, the reference-free methods, which do not need to decipher the community structure of the samples, but are rather dependent on using sample features (i.e., k-mers), have emerged as an alternative approach for sample comparison and searches (Comin et al., 2020). The limitation of such an approach is also apparent, one of which is the weak interpretability of the results. Crowd sourcing might be another way toward the optimal solution of microbiome sample comparison and searches. The MetaSUB challenge (metasub.org) is a good example of citizen science working toward better microbiome sample searches and source tracking. The spike-in of synthetic DNA into the samples is another very promising strategy for accurate source tracking (Qian et al., 2020), yet it needs the combination of both experimental and computational techniques.

What could we do after an accurate sample search? Gene mining, function mining, etc. So many microbiome functional mining tasks have been utilized in the past, and deep learning approaches might be the powerful tool for the way forward (Wang et al., 2019). Apart from the developed algorithms for better microbiome sample comparison and searches, these powerful methods could be applied on the yet-to-be-realized microbiome dark matters. The microbiome dark matter samples that need to be searched include: virome (Paez-Espino et al., 2016), protist (Miao et al., 2020), etc.

Finally, based on the advanced machine learning methods, together with all types of microbiome data, it has become clear that in the area of microbiome research, comparison and searches should not be limited to sample level analysis, but should be extended for other meaningful pattern discoveries. These more diverse sets of patterns that need to be searched include direct functional gene searches (Arango-Argoty et al., 2018; Hannigan et al., 2019), specie-species relationship searches (Vieira-Silva et al., 2016), network patterns, and network motif searches (Jing et al., 2020), etc.

In summary, the development and application of microbiome sample comparison and searches have already advanced to the point that researchers could reliably use them for accurate and fast sample searches. However, this is not the end of the research along this line, but rather provides several more interesting venues for researchers to explore for more in-depth understanding of the microbes and their functions within and across the microbial communities. We foresee that accelerated research on these venues in the near future would yield more exciting results soon.

## Author Contributions

KN conceived and proposed the idea, and designed the study. YZ, HC, and KN contributed to editing and proof-reading the manuscript. All authors read and approved the final manuscript.

## Conflict of Interest

The authors declare that the research was conducted in the absence of any commercial or financial relationships that could be construed as a potential conflict of interest.

## Footnotes

1 https://github.com/HUST-NingKang-Lab/ONN4MST

2 https://www.ebi.ac.uk/metagenomics/biomes

3 http://camda2019.bioinf.jku.at/

4 https://www.epa.gov/sciencematters/microbial-source-tracking-how-did-get-there

5 https://github.com/HUST-NingKang-Lab/Meta-Prism-2.0

## Supplementary Material

Supplementary Table 1 Samples used in assessment of different methods on a standard benchmark dataset.
